# Pain Assessment in INTensive care (PAINT): an observational study of physician‐documented pain assessment in 45 intensive care units in the United Kingdom

**DOI:** 10.1111/anae.13786

**Published:** 2017-02-19

**Authors:** H. I. Kemp, C. Bantel, F. Gordon, S. J. Brett, PLAN, SEARCH, H. C. Laycock, Sohail Bampoe, Carsten Bantel, Mevan Gooneratne, David Highton, Phil Hopkins, Carolyn Johnston, Peter Odor, Harriet Kemp, Helen Laycock, Daniel Martin, James O'Carroll, Sioned Phillips, Anil Visram, Omar Siddique, Edward Burdett, Rosie May, Sonia Renwick, Martin Gray, Michael Spiro, Val Luoma, Holly Chamarette, Trudy Young, Duncan Wagstaff, Henry Lewith, Shan Gowrie, Jon Bramall, Lucy Collison, Josephine Mansell, Kevin Hamilton, Alexander Leigh, Jeremy Dawson, Clare Morkane, Paul Balla, Bhavin Shukla, Francesca Rublotta, Jonathan Cousins, David Magee, Catherine Cashell, Gurleen Kooner, Glenn Arnold, Vanessa Garnelo Ray, Olivia Clancy, Nicole Whitehead, Gurleen Kooner, Jonathan Handy, Marcella Vizcaychipi, Zara Edwards, Melanie Davis‐Hall, Melissa Addy, Shaman Jhanji, Tim Wigmore, Linsey Christie, Lauren Sidon, Munita Grover, Prasan Panagoda, Lara Howells, Charles Cartwright, Richard Doyle, Megan Griffith, Emma Casely, Andrew Holdgate, Stephanie Rich, Maria Henriksson, Sian Jaggar, Kate Tatham, Mirian Kadry, Claire Finlay, Ajoy Pandit, Savviz Mehdipour, Cheng Ong, Heena Bidd, Suneil Ramessur, Sibtain Anwar, Nadia Blunt, Hannah Williams, Chiara Tosini, Alessandra Parini, Ravi Kumar, Victoria Ferrier, Aman Gupta, Aoife Canavan, Matt Dickinson, Ben Carey, Karthik Somasundaram, Samanthi De Silva, Arif Moghulm, Kanika Dua, Barry McHugh, Rachel Chapman, Marcus Peck, Amy Sangam, Hadi Al‐Sahaf, Tharumalingam Gowripalann, Sreekumar Kunnumpurath, Ramy Mottaleb, Kate Fletcher, Milind Bhagwat, Alex Eeles, Annie Hunningher, Finn Nesbit, Danny Turton, Chris Barringer, Peter Dannatt, Venkat Shenoy, Peter Keogh, Bobby Krishnachetty, Fiona Mendes, Janis Ferns, Charles Kennedy, Roxana Sandru, Naush Husain, Liesel Holler, Lucy Barnes, Patrick Thorburn, William Shippam, Sindy Lee, Sara Mahgoub

**Affiliations:** ^1^ Imperial CollegeLondonUK; ^2^ Oldenburg UniversityOldenburgGermany; ^3^ Pan‐London Peri‐operative Audit and Research NetworkUK; ^4^ South‐East Anaesthetic Research ChainUK

**Keywords:** critical care, intensive care unit, pain assessment, pain terms, physicians

## Abstract

Pain is a common and distressing symptom experienced by intensive care patients. Assessing pain in this environment is challenging, and published guidelines have been inconsistently implemented. The Pain Assessment in INTensive care (PAINT) study aimed to evaluate the frequency and type of physician pain assessments with respect to published guidelines. This observational service evaluation considered all pain and analgesia‐related entries in patients’ records over a 24‐h period, in 45 adult intensive care units (ICUs) in London and the South‐East of England. Data were collected from 750 patients, reflecting the practice of 362 physicians. Nearly two‐thirds of patients (n = 475, 64.5%, 95%CI 60.9–67.8%) received no physician‐documented pain assessment during the 24‐h study period. Just under one‐third (n = 215, 28.6%, 95%CI 25.5–32.0%) received no nursing‐documented pain assessment, and over one‐fifth (n = 159, 21.2%, 95%CI 19.2–23.4)% received neither a doctor nor a nursing pain assessment. Two of the 45 ICUs used validated behavioural pain assessment tools. The likelihood of receiving a physician pain assessment was affected by the following factors: the number of nursing assessments performed; whether the patient was admitted as a surgical patient; the presence of tracheal tube or tracheostomy; and the length of stay in ICU. Physician‐documented pain assessments in the majority of participating ICUs were infrequent and did not utilise recommended behavioural pain assessment tools. Further research to identify factors influencing physician pain assessment behaviour in ICU, such as human factors or cultural attitudes, is urgently needed.

## Introduction

Pain is common among patients admitted to intensive care units (ICUs), with a prevalence of 40–77% 1, 2, 3, 4. Failure to conduct appropriate pain assessment hinders adequate pain management, and pain can lead to deleterious acute and chronic physiological and psychological consequences 5. Simply assessing pain can improve patient satisfaction and clinical outcomes, including: number of ‘ventilator days’; length of intensive care stay; and survival 2, 6, 7. This is thought to be due to increased prioritisation of pain and more frequent alterations to analgesic prescriptions 2. Indeed, protocolised analgesic management, reliant on validated and frequent assessment, improves patient outcomes 8.

Evidence suggests that over half of critically unwell patients do not receive regular pain monitoring in the ICU 9. Nursing staff perform the majority of assessments, with little evidence regarding the physicians’ role in this process 10. Regular documentation by physicians of a patients’ history and examination in medical notes forms the basis of patient records. This ensures safe, comprehensive patient handover and continuity of care. Documentation of the assessment of pain in this essential record by physicians is thought to be less frequent than that of other physiological parameters, such as haemodynamic assessments 11.

Pain assessment in ICU is challenging, as the subjective experience of pain requires reliable communication to alert staff to its presence. Communication is, however, often impaired in ICU as a result of sedation, delirium, and disease or due to the presence of iatrogenic airway devices. Consequently, the use of ‘gold‐standard’ self‐assessment tools are not always appropriate. Under these circumstances, staff rely on either subjective, often inaccurate assessments 12, or on unfamiliar but validated behavioural pain scales. To address these challenges, guidelines have been developed in both Europe 13 and the USA 14. The American College of Critical Care Medicine (ACCM) 14 has emphasised the need for regular pain assessment along with the use of validated tools, such as the critical care pain observational tool (CPOT) 15, and the behavioural pain scale (BPS) 16. Despite their dissemination, recent surveys indicate limited use among nursing staff 17, 18. To date, there are no observational data regarding physician pain assessment practice. Rather, the available information has been derived from surveys evaluating recall of practice, largely completed by nursing staff 19, 20. Furthermore, the use of ICU pain assessment guidelines in the UK has not been evaluated.

We aimed firstly to evaluate the frequency and type of pain assessment conducted by physicians in ICUs in London and South‐East of England; and secondly to identify whether pain assessment was documented less frequently than other physiological parameters, and to describe any patient or unit‐specific factors that influenced pain assessment. Finally, we also evaluated nursing assessment and documentation of pain, to identify whether ICUs were meeting recognised standards, and to provide information about the culture of pain assessment for individual ICUs.

## Methods

**Figure 1:**
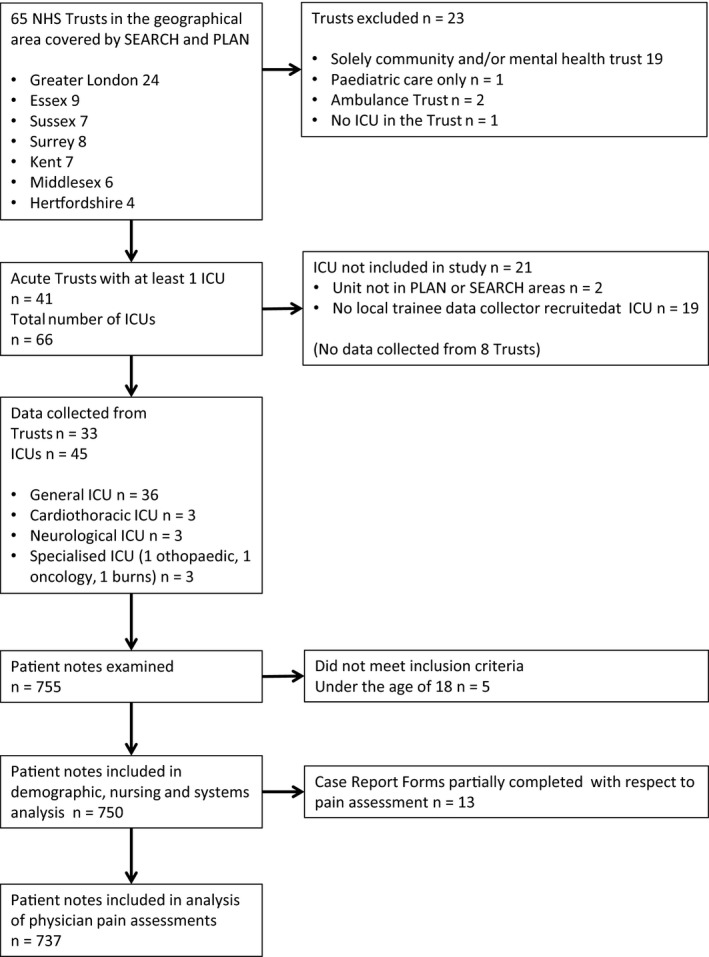
Flow diagram of intensive care unit and patient recruitment. PLAN; Pan‐London Peri‐operative Audit and Research Network; SEARCH; South‐East Anaesthetic Research Chain.

The Pain Assessment in INTensive Care (PAINT) study was a retrospective observational service evaluation conducted by two anaesthetic trainee networks, the Pan‐London Peri‐operative Audit and Research Network (PLAN, www.uk-plan.net) and the South‐East Anaesthetic Research Chain (SEARCH, www.searchkss.co.uk). We audited practice against standards described by the American College of Critical Care Medicine 14. The PLAN and SEARCH networks geographically cover 43 acute NHS trusts across London, Essex, Surrey, Sussex and Hertfordshire, as defined by the Department of Health (Health and Social Care Information Centre, http://www.hscic.gov.uk). Figure 1 shows participating Trusts and ICUs. Under current UK research governance, this study was determined to be a service evaluation, and therefore did not require formal ethical registration or individual patient consent (confirmed through discussion with Research and Development and Clinical Governance departments at the co‐ordinating centre, Chelsea and Westminster Hospital NHS Foundation Trust, London; and by the Health Research Authority decision tool (http://www.hra-decisiontools.org.uk/research/)). The protocol was registered, and appropriate approval granted from audit departments at each individual Trust. Between January and March 2015, individual ICUs identified one weekday 24‐h study period (Monday to Friday), to collect data.

All patients admitted to the ICU aged 18 years or older were included. We reviewed all entries in the patients’ medical notes and observation charts over the 24‐h study period (for data extraction form, see Appendix 1). Any mention of pain in physician‐written medical notes was recorded, including any pain assessment tool that was used. Each physician was allocated an anonymised code, so that individual physician practice could be considered. Drug charts were examined for analgesic and sedative prescriptions. We recorded physician documentation of cardiovascular, respiratory, gastro‐intestinal, genito‐urinary and neurological assessments.

We collected patient‐specific characteristics, including: age, sex; admitting speciality; length of stay in ICU; and whether the patient had an tracheal tube or tracheostomy. Unit‐specific characteristics recorded included: the ICU type (general or specialist); bed occupancy; and format (paper or computerised) for recording observations, prescribing analgesia and recording medical notes.

The number of nursing pain assessments and tools utilised, along with assessment scores, were collected from observation charts and nursing notes. No patient outcome data were collected. Anonymised patient data were collected on paper case report forms before submission to the secure central server via the nhs.net email system.

We analysed the data using IBM SPSS v22.0 and Stata v13.0 (for hierarchical modelling) in collaboration with the statistics service at Imperial College, London. Data were tested for normality (Shapiro–Wilk test). The p values underwent Bonferroni correction to account for multiple comparisons. We performed univariate analysis of variables that influenced the frequency of pain assessments using Mann–Whitney U‐test comparisons for grouped non‐continuous data. Spearman's correlation was used for correlation of continuous measures, and chi‐square testing for categorical group comparisons such as physician grade. Variables identified as important in determining the frequency of physician pain assessments were then tested using hierarchical regression modelling. Because the data had a nested structure (patients were nested within units), the use of hierarchical models was required, since this took into account the variability between units, as well as the correlation between patients within the same unit. The two‐level model identified unit and patient‐specific predictors of the frequency of documented physician pain assessments. Each hospital was coded, and investigators were blinded to patient location. The largest proportion of missing data related to the type of pain assessment used by physicians, representing 22 (5.3%) patient assessments. In 13 (1.7%) patients, the presence or absence of a physician‐documented pain assessment was incomplete, being partially recorded. Therefore, to calculate incidence, we used n = 737 as the denominator. For all other analyses (other than incidences) in these 13 individuals, we imputed that if the data collector had not recorded whether an assessment had taken place on the data extraction form, no assessment had been documented in the medical notes.

## Results

All 45 recruited ICUs submitted patient and hospital data. Data were collected from 755 patients; five were under the age of 18 years and were not studied, leaving a sample of 750 patients.

Baseline characteristics are described in Table 1. Two hundred and twenty‐seven (30.3%) patients were prescribed continuous strong opioid infusions, and 40 (5.3%) were reviewed by a specialised pain service. One hundred and twenty‐five (16.6%) patients received a change to their analgesic prescription during the study period.

**Table 1:** Baseline characteristics. Values are number (proportion) or median (IQR [range])

Patients’ demographics	n = 750
Men	412 (54.9%)
Women	338 (45.1%)
Age, years	64 (50–75 [19–103])
Length of stay, days	3 (1–10 [0–219])
Tracheal tube/tracheostomy	
Yes	285 (38.0%)
No	465 (62.0%)
Admitting speciality	
Medical specialty	353 (47.1%)
General medicine	216 (28.8%)
Respiratory	44 (5.9%)
Haematology/oncology	22 (2.9%)
Neurology	20 (2.7%)
Cardiology	19 (2.5%)
Renal	17 (2.3%)
Infectious diseases	5 (0.7%)
Liver	10 (1.3%)
Surgical specialty	381 (50.8%)
General surgery	146 (19.5%)
Neurosurgery	50 (6.7%)
Cardiothoracic surgery	45 (6.0%)
Orthopaedic	37 (4.9%)
Vascular surgery	24 (3.2%)
Trauma	20 (2.7%)
Gynaecology	19 (2.5%)
Ear nose and throat surgery	14 (1.9%)
Urology	13 (1.7%)
Plastic surgery	7 (0.9%)
Obstetrics	6 (0.8%)
Other (no admitting specialty)	16 (2.1%)

Data were submitted from 45 ICUs and represented a total of 763 available critical care beds (level 2 and level 3). The ICUs were located in either teaching hospitals (29 ICUs, 64.4%) or district general hospitals (16 ICUs, 35.6%). Of the 750 patients, 355 (37.3%) were treated in teaching hospitals, and 395 (52.7%) in district general hospitals. Medical and nursing observations were recorded using electronic systems in 16 ICUs (35.6%), whereas 17 ICUs (37.8%) used electronic drug charts. Specific physician daily ward round proformas were used in 37 ICUs (82.2%), of which six ICUs (13.3%) included a section to record pain assessments.

Data reflect the practice of 362 physicians (in order of seniority: 89 consultants; 133 Specialist Trainees; 76 Core Trainees; 43 Foundation Year doctors; 20 ‘other’; and one missing). A total of 1734 separate patient assessments were documented during the 24‐h study period.

Of the 737 complete patient records, 475 patients (64.5%, 95%CI 60.9–67.8%) had no documentation of pain assessment by a physician. This was higher for patients with either a tracheal tube or a tracheostomy in situ, than for patients with an absence of these airway devices 211, (76.2%, 95%CI 71.4–81.3%) vs. 264, (57.4%, 95%CI 52.8‐61.8%); p < 0.001. Two hundred and sixty‐two patients (35.5%, 95%CI 32.2‐39.1%) had at least one documented pain assessment, as compared with 718 (95.7%, 95%CI 94.0‐97.0%) patients who received at least one documented cardiovascular assessment.

Of the 227 patients receiving continuous opioid infusions, 85 (37.4%) had a physician‐documented pain assessment. One hundred and twenty‐five (16.7%) patients had a change in analgesic prescription recorded on the drug chart, and 54 (43.2%) of these patients had no reference to pain assessment in their medical notes.

Physician experience, as identified by grade of training, did not significantly influence frequency of physician‐documented pain assessments (χ^2^ = 5.00, p = 0.28). Overall, 413 (23.8%) documentation episodes included a pain assessment (146 (22.5%) of consultant, 142 (25.2%) of Specialist Trainee, 81 (27.9%) of Core Trainee, 35 (24.5%) of Foundation Year, and 27 (33.3%) of ‘other doctor’ entries).

**Figure 2:**
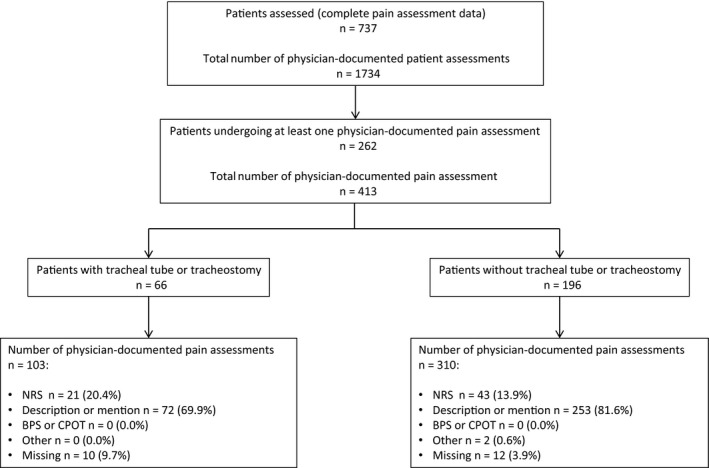
Pain assessment tools used by physicians. NRS, numerical rating scale; BPS, behavioural pain scale; CPOT, critical care pain observation tool. *737 of the 750 patients had complete case report forms regarding physician pain‐assessment documentation.

Figure 2 outlines the types of pain assessment tools recorded in the medical notes for patients with and without a tracheal tube or tracheostomy. No behavioural pain assessment tools were recorded in the physician notes. Pain assessments were most likely to be recorded by physicians during ward rounds and daily reviews (355 (86.0%) pain assessments). However, out of a total of 851 ward rounds, 689 (81.0%) did not include references to analgesia or pain relief, and there was no difference in documentation with the use of electronic or paper charts (p = 0.683), or proformas with prompts for pain assessment (p = 0.669).

Table 2 shows the comparisons of the median number of cardiovascular and other organ system assessments, vs. the median number of pain assessments. This revealed a significantly reduced prevalence of the latter.

**Table 2:** Comparisons of the number of pain assessments with the number of other system assessments. Values are median (IQR[range])

System	Number of assessments documented per patient in 24 h	p value
Cardiovascular	2.0 (1.0–3.0 [0.0–5.0])	< 0.001
Respiratory	2.0 (1.0–3.0 [0.0–5.0])	< 0.001
Gastro‐intestinal	2.0 (1.0–2.0 [0.0–13.0])	< 0.001
Genito‐urinary	2.0 (1.0–2.0 [0.0–4.0])	< 0.001
Neurology	2.0 (1.0–2.0 [0.0–4.0])	< 0.001
Pain	0.0 (0.0–1.0 [0.0–4.0])	NA

Bonferroni corrected p value for multiple comparisons = 0.0083.

Two‐hundred and fifteen patients (28.7 95% CI 25.5–32.0%) had no documented nursing pain assessment. The median (IQR [range]) number of nursing pain assessments per patient was 2.0 (0.0–5.0 [0.0–24.0]). Three‐hundred and sixteen patients (42.1%, 95%CI 38.6–45.7%) received fewer than two documented pain assessments during the 24‐h study period. This equates to less than one documented pain assessment every 12‐h nursing shift. Of the 227 patients prescribed strong opioids, 78 (34.4%, 95%CI 28.5–40.8%) did not receive a documented nursing pain assessment. Two of the 45 ICUs used behavioural assessment tools for nursing pain assessment, with the commonest assessment tool being a 0–3 scale (Table 3). In total, 159 patients (21.6%, 95%CI 18.8–24.7%) received neither nursing nor physician documentation of pain assessment.

**Table 3:** Types of pain assessment tool used for nursing pain observation. Values are number (proportion)

Pain assessment tool	Total number of patients n = 750	Number of patients with tracheal tube or tracheostomy n = 285
No score	215 (28.6%)	108 (37.9%)
NRS	95 (12.7%)	22 (7.7%)
Description	101 (13.5%)	55 (19.3%)
BPS	4 (0.5%)	3 (1.1%)
CPOT	8 (1.1%)	6 (2.1%)
0–3	266 (35.5%)	77 (27.0%)
Other	59 (7.9%)	13 (4.6%)
Missing	2 (0.2%)	1 (0.4%)

NRS, numerical rating scale; BPS, behavioural pain scale; CPOT, critical care pain observation tool.

To identify patient and unit‐specific variables for hierarchical modelling, we first undertook univariate analysis. Table 4 includes variables and their p values; those that approached statistical significance were taken forward in further modelling.

**Table 4:** The influence of patient and unit‐specific variables on frequency of physician pain assessments

Variables tested	p value
Patient‐specific characteristics	
Sex – male vs. female	0.345
Age	0.039
Length of stay on ICU	< 0.001
Tracheal tube/tracheostomy – present vs. absent	< 0.001
Admitting specialty – medical vs. surgical	< 0.001
Strong opioid – prescribed vs. not prescribed	0.909
Number of nursing pain assessments	< 0.001
Unit‐specific characteristics	
Unit size (number of beds)	0.837
Bed occupancy	0.772
Observation charts – paper vs. electronic	0.683
Medical notes – paper vs. electronic	0.734
Pain prompt on ward round proforma – present vs. absent	0.658
ICU type – general vs. specialist	< 0.001
Hospital type – district general vs. teaching	< 0.001

The number of documented physician assessments of pain was used as an outcome measure for subsequent hierarchical modelling. Since this outcome was skewed, a Poisson distribution was assumed. Patient characteristics tested were: age; length of stay on ICU; presence of airway device; admitting speciality (medical or surgical); and the number of documented nursing reviews. Unit‐specific characteristics tested were ICU type (general or specialised) and hospital type (district general or teaching). These results are shown in the Table 5.

**Table 5:** Characteristics showing a significant effect on frequency of physician pain assessment

Explanatory variables	Estimated effect	Standard error	Incidence rate ratio^a^	p value
Length of stay in specialised units	0.002	0.006	1.0002	0.026
Length of stay in general units	−0.011	0.005	0.989	0.038
Surgical vs. medical patients without an airway device	0.314	0.149	1.36	< 0.001
Surgical vs. medical patients with an airway device	0.023	0.183	−10.2	NS
Nursing pain review	0.060	0.014	1.06	< 0.001
Specialised vs. general unit^b^	0.578	0.360	1.76	NS

a Incidence rate is the rate at which the number of pain assessments occur.

b This comparison is for length of stay set to its average.

Length of stay had a significant effect on pain assessment, but the effect depended on whether the ICU was general or specialised. For specialised ICUs, as the length of stay increased, so did the number of pain assessments. For a unit increase in number of days in a specialised ICU, the number of pain assessments performed by physicians increased by a factor of 1.003 (p = 0.026). This was in contrast to general ICUs, where the number of pain assessments performed by physicians was reduced by a factor of 0.988 (p = 0.038).

Surgical patients without a tracheostomy or tracheal tube had a higher number of physician pain assessments performed when compared with medical patients without a tracheostomy or tracheal tube (p < 0.001). However, for those patients with an airway device present, this difference was not significant. In the final model, patient age did not have an effect on pain assessment.

The number of documented nursing assessments significantly influenced the number of documented physician assessments (p < 0.001). An increase by one of the number of nursing assessments was associated with an expected increase in physician assessments by a factor of 1.061.

The only unit‐specific factor that showed a significant effect on physician‐documented pain assessment was whether the unit was a general or specialised ICU. However, as described previously, this effect interacted with patient length of stay. The hospital type (district general or teaching), had no effect on pain assessment. Although unit and patient‐specific variables helped to explain inter‐unit variability, there was a proportion of unexplained variability, with an estimated effect of 0.771 (0.433–1.371).

## Discussion

Nearly two‐thirds of the patients included in this study did not have pain assessments documented by physicians. The absence of documentation does not equate necessarily with the absence of an assessment. However, the failure to record examination findings at best reflected the fact that pain documentation was afforded a low priority, and at worst, that no assessment of pain was undertaken by the medical staff. Documentation of the effects of analgesic medication, and the reasons for changes in pain prescriptions are integral to continuity of patient care. Over three‐fifths of patients receiving opioid infusions, and over two‐fifths of patients who received changes in analgesic medication lacked a physician documentation of pain assessment. Consequently, drug effects and the rationales behind pain medication changes were not transparently communicated in the medical notes. It was notable that over four‐fifths of ward round reviews failed to record pain assessments, again implying that communication of this aspect of patient care was lacking.

This was in stark contrast to the frequency of documentation of other physiological systems, such as cardiovascular observations, that were routinely recorded in the majority of physician entries in the medical notes. Therefore, it is unlikely that an absence of documentation of pain assessment reflected poor medical documentation in general. The routine presence of cardiovascular observations in medical notes may have been due to a higher confidence by physicians in parameters recorded using medical devices, which provided recognisable quantitative data. In contrast, the more subjective data produced from an observational pain assessment could have been less familiar to treating physicians, and something in which they had less faith. The consistency with which cardiovascular and respiratory observations were documented when compared with those of pain, could highlight the low priority given to pain as a problem requiring physician attention, or that more patient‐centred variables are seldom documented by physicians.

There is a need for these factors to be explored and modified, to identify whether a culture of improved pain assessment by all healthcare professionals also improves patient outcome.

Reasons as to why physicians do not record observations about pain in medical records are unclear, although it is likely that human factors play a role. The UK Health and Safety Executive has classified influences on healthcare performance into job or task factors, and as either individual and organisational factors 21. Pain assessments could have been affected by all of these categories. Medical professionals failed to utilise correct tools to perform the task, potentially due to lack of knowledge, lack of time or the perceived complexity of the assessment. Individual factors could have included a lack of motivation to undertake the assessment; for example, due to a perception that sedated patients do not experience pain, work overload, or a lack of competence in using the assessment tools. Importantly, our data demonstrates that this was a phenomenon among physicians that was unaffected by their level of experience, and that interventions need to be targeted at all levels of physician seniority.

Finally, organisational factors might have included a lack of clarity as to whose role it was to perform pain assessments. Physicians may have assumed that pain assessment was a nurse‐led role (yet there was a positive relationship between nursing and physician assessment); poor communication between nursing staff and physicians, and a culture where pain assessment was routinely ignored may have further influenced performance.

It is difficult to fully explain why length of stay influenced physician pain documentation positively in specialist units, but had the opposite effect in general units. This could reflect the fact that in specialist units discharge from ICU might be delayed due to recognition of an issue with pain management, whereas this may not occur in a general unit. Conversely, in general hospitals, it is more likely that step‐down care and discharge from ICU may be delayed due to a shortage of normal ward beds. Therefore, patients who do not require a high level of care remain in the ICU where it is perceived that, because they are less unwell, they require less frequent pain assessment. However, patients in general wards should also be regularly assessed for pain 22, and this should not be a reason for an association with length of stay.

Our findings demonstrate that medical patients without tracheal tubes or tracheostomies received fewer pain assessments than surgical patients. This is despite evidence indicating that medical ICU patients report higher pain scores when compared with surgical patients 1. Furthermore, over three quarters of patients with an airway device in situ failed to have a pain assessment documented. It is unlikely that patients whose tracheas were intubated did not experience pain, as the literature suggests that even simple procedures such as suctioning endotracheal tubes, are painful 23. These findings reflect the challenges in evaluating pain in those unable to self‐report, and it is worrying that behavioural pain scales were so poorly adopted by all healthcare professionals in this study.

Internationally, widespread failure of the adoption of frequent validated pain assessments by nurses has been reported 17, 18, despite the publication of the ACCM guidelines 14. Our data replicate these findings in a large sample of UK ICUs. However, our results demonstrate that this is more pronounced in physicians than nurses, and was emphasised by the complete absence of the inclusion of behavioural pain assessment tools in patients’ medical records. A ‘0–3’ score was most frequently employed, and it is unclear what this score represented; it might have been an observed version of a self‐report measure, the verbal rating scale (VRS) 24, a tool that has not been validated for use in critical care. The lack of adherence to the ACCM guidelines is concerning, as regular, validated pain assessment has consistently been associated with improved patient outcomes 6, 8.

Possible reasons for the gap between recommendation and implementation include a lack of knowledge, and staff scepticism regarding the benefits of such behavioural tools 25. Recent work by Van der Woude et al. 18 highlighted the belief among nurses that subjective nursing assessment of pain is superior to validated scales, despite evidence to the contrary 26.

Interestingly, an increase in the frequency of nursing pain assessments was associated with an increase in the frequency by physicians. Certainly, some units stood out for prioritising pain assessment, and further qualitative or ethnographic work is required to identify why these units assessed pain more frequently and robustly than others.

Limitations of our study include the use of a short observation period, rather than a longitudinal approach. We also used an independent review of documentation as a proxy for bedside review of pain by a physician. A further limitation is that retrospective data collection may fail to capture real‐time changes. However, much of the published literature on pain in ICU has relied on the recall of practice by healthcare professionals using surveys, and so our method adds to the evidence base in a robust way. We recruited ICUs within a specific geographical region, many of which engage in regular audit and research. It is, therefore, unclear how generalisable these results are to practice elsewhere; however, evidence from nursing literature shows that pain is managed poorly in ICUs in many countries 17, 18. This study did not address procedural pain; a recent trial of the management of procedural pain highlighted unacceptably high baseline pain scores, suggesting that there was a need to control background pain before adequate procedural pain therapy can be attempted 27. Finally, as a service evaluation study, we did not explore whether pain assessments influenced patient outcomes. We designed the study to focus on the unpublished frequency of physician pain assessment and documentation, instead of the already established effects on patient outcome.

This study shows that pain assessment is not being delivered in a reliable manner across ICUs in the UK, mirroring similar problems with ICU pain assessment worldwide. Although ICUs in the UK are well funded, and have good clinical outcomes in terms of survival, for example after sepsis 28, we have shown that there is room for improvement regarding more patient‐centred elements such as pain management. The barriers to physician involvement in pain management need to be explored and modified. Evaluation of these changes needs to identify whether a culture of improved pain assessment in all healthcare professionals can also impact on short and longer term patient outcomes.

This study adds to evidence that pain assessments conducted in intensive care are inconsistent in both frequency and method of delivery. We have demonstrated that both medical and nursing staff fail to document pain assessments, despite an association with improved patient outcomes. There is an urgent need for ethnographic research exploring pain assessment in intensive care, and qualitative studies to identify reasons for the lack of prioritisation of pain and adoption of guidelines.

## Appendix 2 List of named PLAN and SEARCH contributors and affiliated institutions

PLAN Core Committee for PAINT: Sibtain Anwar (Guys Hospital, London and St Thomas’ Hospital, London), Sohail Bampoe (St Georges Hospital, London), Carsten Bantel (Chelsea and Westminster Hospital, London), Mevan Gooneratne (The Royal London Hospital, London), David Highton (University College London Hospitals) Phil Hopkins (Kings College Hospital, London), Carolyn Johnston, Peter Odor (St Georges Hospital, London), Harriet Kemp, Helen Laycock (Imperial College, London), Daniel Martin (Royal Free Hospital, London), James O'Carroll (The Royal London Hospital, London), Sioned Phillips (Croydon University Hospital, Croydon), Anil Visram (The Royal London Hospital, London).

SEARCH Core Committee for PAINT: Omar Siddique (Medway Maritime Hospital, Gillingham).

PLAN and SEARCH Site Investigators are: Edward Burdett, Rosie May (University College London Hospitals), Sonia Renwick, Martin Gray, Michael Spiro (Royal Free Hospital, London), Val Luoma, Holly Chamarette (National Hospital for Neurology and Neurosurgery, London), Trudy Young, Duncan Wagstaff, Henry Lewith (The Whittington Hospital, London) Shan Gowrie, Jon Bramall, Lucy Collison, Josephine Mansell (The Lister Hospital, Stevenage), Kevin Hamilton, Alexander Leigh (The Princess Alexandra Hospital, Harlow) Jeremy Dawson, Clare Morkane (Barnet Hospital, Barnet), Paul Balla, Bhavin Shukla (Royal National Orthopaedic Hospital, Middlesex), Francesca Rublotta (Charing Cross Hospital, London), Jonathan Cousins, David Magee, Catherine Cashell, Gurleen Kooner (Hammersmith Hospital, London), Glenn Arnold, Vanessa Garnelo Ray, Olivia Clancy, Nicole Whitehead, Gurleen Kooner (St Marys Hospital, London), Jonathan Handy, Marcella Vizcaychipi, Zara Edwards, Melanie Davis‐Hall, Melissa Addy (Chelsea and Westminster Hospital, London), Shaman Jhanji, Tim Wigmore, Linsey Christie, Lauren Sidon (The Royal Marsden Hospital, London), Munita Grover, Prasan Panagoda, Lara Howells, Charles Cartwright (Northwick Park Hospital, London) Richard Doyle, Megan Griffith (Central Middlesex Hospitals, London), Emma Casely, Andrew Holdgate, Stephanie Rich, Maria Henriksson (Hillingdon Hospital, London), Sian Jaggar, Kate Tatham (The Royal Brompton Hospital, London), Mirian Kadry, Claire Finlay (West Middlesex Hospital, London), Ajoy Pandit, Savviz Mehdipour (Watford General Hospital, Watford), Cheng Ong, Heena Bidd, Suneil Ramessur, Sibtain Anwar, Nadia Blunt (Guys Hospital, London and St Thomas’ Hospital, London), Hannah Williams, Chiara Tosini, Alessandra Parini (St Georges Hospital, London), Ravi Kumar, Victoria Ferrier, Aman Gupta, Aoife Canavan (East Surrey Hospital, Redhill), Matt Dickinson, Ben Carey (Royal Surrey County Hospital, Guildford), Karthik Somasundaram, Samanthi De Silva (St Peters Hospital, Chertsey), Arif Moghulm, Kanika Dua, Barry McHugh, Rachel Chapman (Croydon University Hospital, Croydon), Marcus Peck, Amy Sangam (Frimley Park Hospital, Frimley), Hadi Al‐Sahaf, Tharumalingam Gowripalann (Kingston Hospital, Kingston upon Thames), Sreekumar Kunnumpurath, Ramy Mottaleb, Kate Fletcher (St Helier Hospital, Carshalton), Milind Bhagwat, Alex Eeles (Epsom Hospital, Epsom), Annie Hunningher (The Royal London Hospital, London), Finn Nesbit, Danny Turton (Homerton University Hospital, London), Chris Barringer, Peter Dannatt (Whipps Cross University Hospital, London), Venkat Shenoy, Peter Keogh (Basildon University Hospital, Basildon), Bobby Krishnachetty, Fiona Mendes (Southend University Hospital, Westcliff‐on‐Sea), Janis Ferns (Broomfield Hospital, Chelmsford), Charles Kennedy (Medway Maritime Hospital, Gillingham), Roxana Sandru (Queen Elizabeth The Queen Mother Hospital, Margate), Naush Husain (Tunbridge Wells Hospital, Pembury), Liesel Holler (Darent Valley Hospital, Dartford), Lucy Barnes, Patrick Thorburn (Worthing Hospital, Worthing), William Shippam (Princess Royal Hospital and Hurstwood Park Hospital, Haywards Heath), Sindy Lee, Sara Mahgoub (William Harvey Hospital, Ashford).
